# Cognitive Radio Networks: Technologies, Challenges and Applications

**DOI:** 10.3390/s25041011

**Published:** 2025-02-08

**Authors:** Yang Xu, Kechen Zheng, Xiaoying Liu, Zhao Li, Jia Liu

**Affiliations:** 1 School of Computer Science and Technology, Xidian University, Xi’an 710071, China; yxu@xidian.edu.cn; 2 School of Computer Science and Technology, Zhejiang University of Technology, Hangzhou 310023, China; xiaoyingliu@zjut.edu.cn; 3 School of Cyber Engineering, Xidian University, Xi’an 710071, China; zli@xidian.edu.cn; 4 Center for Strategic Cyber Resilience Research and Development, National Institute of Informatics, Tokyo 101-8430, Japan; jliu@nii.ac.jp

## 1. Introduction and Current Trends in the Field

In recent years, Cognitive Radio Networks (CRNs) have emerged as a transformative solution to address the growing demand for wireless spectrum resources and the inefficiencies of traditional static spectrum allocation policies. With the proliferation of wireless devices and the rapid expansion of Internet of Things (IoT) ecosystems, the scarcity of available spectrum resources has become a critical bottleneck. Cognitive Radio, empowered by dynamic spectrum management and intelligent decision-making capabilities, offers a promising way of maximizing spectrum utilization while minimizing interference [1,2,3]. This Special Issue brings together cutting-edge research and innovative solutions aimed at advancing CRN technologies, addressing existing challenges, and exploring diverse applications across industries.

One of the most prominent trends in CRNs is the integration of Machine Learning (ML) and Artificial Intelligence (AI) techniques to enhance spectrum sensing, access, and management. AI-driven models are being employed to predict spectrum availability, optimize resource allocation, and detect unauthorized spectrum usage with improved accuracy [4,5]. Additionally, deep learning approaches are being increasingly utilized for anomaly detection, cognitive decision-making, and dynamic adaptation to rapidly changing wireless environments [6].

Another significant trend is the integration of CRNs with emerging wireless paradigms such as Software-Defined Networking (SDN) and Network Function Virtualization (NFV) [7]. These technologies enable centralized control and programmable network architectures, facilitating seamless coordination and dynamic resource allocation in large-scale CRN applications. Furthermore, the use of blockchain technology is gaining traction in CRNs to ensure secure and transparent spectrum trading and management [8].

CRNs are also witnessing significant progress in addressing coexistence challenges with Primary Users (PUs) and other Secondary Users (SUs). Techniques such as cooperative spectrum sensing and interference management are being refined to minimize Age of Information (AoI) performance and optimize spectrum-sharing mechanisms [9]. Energy efficiency remains a crucial focus area, with researchers exploring low-power spectrum sensing and access techniques to extend the operational lifespan of battery-constrained cognitive devices.

Finally, the application domains of CRNs are expanding beyond traditional telecommunications into sectors such as public safety, disaster recovery, smart cities, and industrial IoT [10,11]. These applications require robust, reliable, and highly adaptive communication networks, and CRNs are increasingly viewed as a key enabler in these scenarios. As we move forward, the convergence of CRNs with other advanced technologies, such as edge computing and terahertz communication, will further unlock their potential to address the ever-growing demands of future wireless ecosystems [12].

In summary, the field of CRNs continues to evolve with the integration of advanced technologies, the exploration of new applications, and the growing focus on security and privacy. We believe the articles in this Special Issue will serve as a valuable resource for researchers, practitioners, and policymakers working toward the advancement of wireless communication systems.

## 2. Scope of Special Issue and Contributions

This Special Issue on “Cognitive Radio Networks: Technologies, Challenges and Applications” aims to collate and disseminate state-of-the-art research advances in the analysis, design, optimization, implementation, and standardization of Cognitive Radio Networks. As a result of the open call for papers, papers related to ML, neural networks, and energy harvesting have been accepted after a rigorous peer-review process and assessed for their technical merit and relevance. The accepted papers cover applied issues in the following fields:

- Spectrum sensing, spectrum sharing, and spectrum learning and prediction;
- Machine learning techniques for Cognitive Radio Networks;
- Energy-harvesting Cognitive Radio Networks;
- Challenges and issues in designing Cognitive Radio Networks and communications;
- High-order cumulants in Cognitive Radio Networks.

These contributions are outlined in more detail below.

In contribution 1, Cervantes-Junco et al. proposed a decision-making algorithm with geographic mobility (DMAGM), which considerably reduces latency and computational complexity, to improve the communication performance of CRNs. They further proposed an improved version of the DMAGM, i.e., a feedback decision-making algorithm with geographic mobility (FDMAGM), where the feedback of the output is incorporated into the algorithm so that it can continually readapt based on the received feedback. In practical scenarios, the DMAGM improves the latency performance, and the FDMAGM improves accuracy and stability.

In contribution 2, Luo et al. employed a spectrum-based encoder to transform unstructured data into the latent space and presented a partial privacy-preserving framework that utilizes the designed interpretable model to restrict access for undesired task-independent attacks while preserving the utility of target tasks. Evaluations on multiple standard datasets show that the framework achieves competitive tradeoffs between privacy and utility.

In contribution 3, Dgani et al. employed a feature-based method that utilizes high-order cumulants (HOCs) as key features for automatic modulation classification (AMC) and explored the best classification feature for each modulation in CRNs. The proposed method performs well with different signal-to-noise ratios (SNRs) and channel conditions and offers a cost-effective and high-quality solution for AMC in various civilian and military applications.

In contribution 4, Rugini et al. analyzed the performance of centralized cooperative spectrum sensing, where SUs collaboratively detect the presence or absence of a primary signal based on compressed measurements, and obtained simplified closed-form expressions that calculate the number of sensors, number of samples, compression ratio, and SNR as a function of the detection probability and false alarm probability. The obtained results have both high accuracy and low complexity and are useful for applications with few sensors and low sample sizes.

In contribution 5, Kim et al. proposed a multitask learning-based deep signal identification network that addresses the deep signal identification problem in advanced spectrum sensing systems for CRNs. Based on performance investigations in terms of task correlation, and model size and computational efficiency evaluations, the comprehensive results emphasize the potential and practical utility of the proposed network for solving deep signal identification problems.

In contribution 6, Zhang et al. proposed a one-timescale iteration-adjustable unlicensed spectrum allocation algorithm where the step size and timescale parameter can be jointly adjusted, and they further proposed a two-timescale iteration-adjustable joint frequency selection and frequency allocation algorithm depending on slow-changing statistical channel state information. Moreover, the advantages of the proposed algorithms were validated in terms of the derived convergence conditions of the two-timescale algorithm and the derived upper bound of the corresponding convergence error.

In contribution 7, Wang et al. analyzed the fourth-order cyclic cumulant (FOCC) of signals with different modulation modes for a narrow-band wireless communication system and proposed an adjacent-frequency interference detection algorithm according to the FOCC of the received signal. The performance of the proposed algorithm in terms of correct rate and computational complexity was analyzed and compared with traditional methods. In a rail transit wireless communication scenario, the proposed algorithm overcomes the Doppler effect and works well at medium SNRs.

In contribution 8, Yu et al. proposed an environment-aware robust algorithm that employs an occasional small packet and designs a rate-adaptive scheme for the IEEE 802.11 standard. The oscillation detection mechanism in the proposed algorithm effectively suppresses abnormal rate fluctuations and further improves system throughput. Compared with similar methods, the proposed algorithm improves the throughput performance of wireless networks in mobile environments.

Contribution 9 is a review article that focuses on Cognitive Radio technology and its applications in the field of civil aviation. Zheng et al. examined the current state of Cognitive Radio technology, including ongoing research and development efforts, regulatory issues, and potential challenges to widespread adoption, and they explored potential applications of Cognitive Radio technology in civil aviation by enabling the intelligent monitoring and management of radio signals.

In contribution 10, based on the energy-harvesting cognitive Internet of Things model, Jiang et al. studied the free-riding problem of SUs and established a penalty mechanism to stimulate SUs to sense the spectrum normally during the sensing process. To improve spectrum utilization, they proposed a two-layer game-based cooperative spectrum sensing and access method that dynamically coordinates the strategies of SUs, and this significantly improved the overall throughput compared with traditional algorithms.

In contribution 11, Molina-Tenorio et al. considered a multilayer perception in a centralized CRN to monitor a multiband spectrum in real time and implemented it in a real wireless communication environment. The results show that the proposed CRN accurately locates PUs with the characteristic bandwidth, carrier frequency and power, avoiding the hidden terminal problem.

In contribution 12, Zhang et al. proposed a buffer-aided relay selection scheme based on deep Q-learning that uses neural networks to fit the Q-function, and they verified the reliability and security performances of the proposed scheme in terms of the connection outage probability and secrecy outage probability. Monte Carlo simulation results demonstrate that the proposed scheme achieves reliable and secure communications compared with similar schemes when applied in two-hop wireless relay networks.

In contribution 13, to solve the problem of vehicles not being detected in dense scenes, Wang et al. developed a new vehicle detection model by adding a normalization-based attention module to the classical YOLOv5s model, and they proposed a real-time small-target vehicle-tracking method that embeds the feature extraction process into the prediction head for training. Adding the spatial attention mechanism and channel attention mechanism module into the proposed model improves the detection accuracy for vehicles in real-time, enhances efficiency, and promotes in-depth research in the fields of vehicle detection and tracking.

## 3. Conclusions

As Guest Editors, we are very pleased with the final outcome of this Special Issue (SI) and look forward to seeing fellow researchers and members of the scientific community enjoy reading the articles within. Furthermore, we would like to extend our special thanks to the managing team of the Sensors journal for their continuous effort and support throughout all stages of editing this Special Issue, including the initial preparation and planning, as well as the submission and review processes for all candidate manuscripts. Finally, we are honored to have received outstanding research papers from the contributing authors and deeply appreciate the reviewers for their assistance, timely feedback, and valuable suggestions.
